# Thinking global health from the perspective of anthropology

**DOI:** 10.1186/s41256-021-00233-z

**Published:** 2021-12-02

**Authors:** Ruobing Ji, Yu Cheng

**Affiliations:** School of Sociology and Anthropology, Sun Yat-Sen University, Guangzhou, Guangdong Province China

**Keywords:** Global health, Medical anthropology, Holism, Political economy of inequality, Cultural diversity, Cultural adaptation, Multispecies ethnography

## Abstract

COVID-19 has brought about political, economic, cultural, and interspecies problems far from medical areas, which challenges academia to rethink global health. For holism principle, anthropology offers valuable insights into these health issues, including the political economy of inequality, cultural diversity, and cultural adaptations, as well as the study of multispecies ethnography. These perspectives indicate that unequal political and economic systems contribute to health problems when people acknowledge disease and illness mechanisms. Moreover, cultural diversity and cultural adaptation are essential for providing appropriate medical solutions. Lastly, as a research method of studying interspecies relationships, multispecies ethnography promotes one health and planetary health from the ultimate perspective of holism. In conclusion, global health is not only a bio-medical concept but also involves political economy, culture, and multispecies factors, for which anthropology proffers inspiring theories and methods.

## Background

The globalization of the world has made human beings one community. Not only that, COVID-19 has heightened awareness that every living thing in this community shares a shared destiny, for which global health is of paramount importance. Nevertheless, how do we define global health? Richard Horton, editor-in-chief of The Lancet, wrote that global health is “an attitude” [1]. It is an attitude and way of looking at life. Moreover, medical anthropologists contribute to this attitude and way by the general principle of anthropology, holism, which simultaneously emphasizes multidimensional humans as social beings and biological beings. More specifically, thinking about global health from the perspective of anthropology starts from three points: the political economy of inequality, cultural diversity and cultural adaptation, and multispecies ethnography.

## The political economy of inequality

How might we detect health problems at a global level? The history of global health, according to this perspective, is one of the unequal relations, dominance, and wealth extraction [2]. As a holistic study, anthropology notices that inequality becomes one of the most critical obstacles to improving global health, which is more than just a biological issue. For thousands of years, the concept of global health was intertwined with imperial ambitions, international politics and commerce, which inspired reformers in the Roman Empire to standardize ditches and sewers in an attempt to manage plagues across provinces—measures that, at least in their view, covered the vast majority of the known world at the time [3]. Peter Brown’s anthropological research in Sardinia also demonstrated malaria in 1907 was associated with inequity in land distribution [4]. It can be seen that social factors like economic status, public administration and education level can all impact global health. Furthermore, the unequal political and economic system among or in different areas has disadvantaged marginalized groups, which exacerbates and maintains health inequity worldwide. To address global health problems, scholars and policymakers should notice the political economy of inequality.

The anthropological research by Paul Farmer in Haiti examined the link between disease and the political economy of inequality [5]. He presented the concept of structural violence in this case, which means that disease results from historically established processes and forces, which are generally economic and political problems caused by western colonists. Through a nuanced portrait of everyday life, the anthropological work investigates how such forces and processes operate together to constrain individual agency. For many less-developed areas, the suffering of racism, sexism, political violence, and poverty, brought by invaders and finally internalized as national systems, limits their choices.

A second example comes from Vikram Patel and Arthur Kleinman’s research on the relationship between poverty and mental disorders [6]. Structural violence leaves the poor vulnerable to mental distress and many without access to mental health services due to poverty, further increasing their mental disorders. Patel and Kleinman concluded that experiences of insecurity and despair, rapid social change, and the risk of violence and physical illness make the poor vulnerable to common mental disorders.

Taking the anthropological view of the political economy of inequality, some diseases like AIDS are but another misfortune that has been added to layers of suffering accumulated historically. Thus, to promote global health, it is necessary to examine which political and economic forces in everyday life are influencing patterns of material deprivation and disease and the relationship between the two, which can help scholars and policymakers conscious of the reform of political and economic systems and the role of social development in realizing health equity.

## Cultural diversity and cultural adaptation

This view explains how a global health system can be appropriately established. Holding the principle of holism, anthropology stresses culture, of which one most crucial point is cultural diversity. Thus, global health is a cultural issue involving every ethnic group and individual living on this planet. Therefore, the global health system should be established under local cultural considerations, that is, based on the local cultural values and realities.

However, tropic medicine, of which the service object is western colonists, historically leaves the existing medical and health governance system centered on western culture. The foundation is the western concept of a global body and society, emphasizing modern medication, surgery, and other quick treatment methods available in western medicine. For example, the maternal mortality rate among poor indigenous people in rural Peru is exceptionally high. Because the health care system brought about by western societies failed to adapt to local culture, including beliefs, norms, and values related to childbirth, and cultural conflicts increased healthcare providers’ discrimination of certain ethnic groups, resulting in their little access to western care services.

In an invited lecture at China Yunnan University in 2014, Judith Farquhar proposed that global health is airdrop medicine, which is insufficient to adapt to local culture. The Bill and Melinda Gates Foundation and other western NGOs airdrop modern biomedicine, including medical technology and medicine, medics and nurses, and medical students, to less-developed areas, spending many materials and human resources to provide medical support. Locals, however, may not use them because of different cultural values or contradictory cultural meanings of medical treatments. As a result, the global health system does not adapt well to the locals it wants to help, resulting from mainly ignoring the culture of diseases and illnesses in the local context.

Global health would be deficient in cultural adaptation if it assumed one cultural context and one universal medical practice. By contrast, following the principle of holism, the anthropological approach—ethnography—meticulously interprets how local beliefs, norms, and values operate on diseases and their treatment in different societies, which contributes to designing a set of culturally adaptable tools that can be appropriate to promote global health.

Anthropology provides research perspectives and approaches that incorporate or increase the weight of local culture in global health policy toolkits, which have previously been largely disregarded in airdrop health. So, how to promote cultural adaptation is a public policy process that should be discussed between with anthropologists and health policy scientists.

## Multispecies ethnography

As the anthropological approach, ethnography has its advantage in long-time studying a group of people’s everyday life, but most of the precious work is anthropocentric and human-centered. Global health can focus on not only humans but also every living thing on Earth. Fifteen years ago, some scientists and public health specialists prompted the “One Health” initiative underlining human-nonhuman entanglements [7, 8]. Anthropology is not the forerunner to care about One Health but proffers an analytic tool—multispecies ethnography—to capture power relations between humans and nonhumans in a more radical way.

In her salmon ethnographic research, Marianne Lien provides a compelling example of how salmon farming is reshaping human societies along with the environment, food, capital, and labor [9]. In this view, the process in which salmon are domesticated by the modern farming industry, are calculated and manipulated as biomass, are packaged from the migration as scalable goods, and are proven by animal NGOs to be sentient species worthy of protection, expresses the unequal power relationship between humans and animals, and constitutes dangerous domestication continuously changing human society.

In some sense, anthropology’s efforts aim not only to expand themes of well-being to other species but also to awaken the moral consciousness of positionality between humans and nonhumans [7]. It means that the anthropological analysis of One Health reminds global health experts of responsibilities, which are not only about human actions but also about transcending species hierarchies in far more holistic and subtler ways. As an anthropological approach, multispecies ethnography is particularly valuable in detecting and interpreting those species hierarchies.

## Conclusions

Anthropology provides global health research with valuable theories and methods based on the principle of holism. The political economy of inequality critically reveals the most profound social cause of global health sufferings and the way to global health equity. Cultural diversity and cultural adaptation meticulously illustrate the role of local context in proving global health support and the urgency of culturally appropriate tools. Multispecies ethnography advances the perception of human-nonhuman positionality and One Health. To sum up, the prosperous engagement of anthropology in global health replenishes the arsenals of theory and methods to promote well-being across political, economic, cultural, and species borders.

## Data Availability

Not applicable.
